# A novel robot-assisted knee arthroplasty system (ROSA) and 1-year outcome: A single center experience

**DOI:** 10.1097/MD.0000000000035710

**Published:** 2023-10-20

**Authors:** Yunus Demirtas, Abdulsamet Emet, Gokhan Ayik, Gokhan Cakmak, Ertugrul Aksahin, Levent Celebi

**Affiliations:** a Yuksek İhtisas University Medical School Orthopedics and Traumatology Department, Ankara, Turkey; b Department of Orthopedics and Traumatology, Etlik City Hospital, Ankara, Turkey; c Ankara Liv Private Hospital, Ankara, Turkey.

**Keywords:** arthroplasty, knee, robotic surgery, ROSA

## Abstract

**Background:**

Total knee arthroplasty is a successful procedure in the treatment of knee osteoarthritis. Searches in surgical technique have focused surgeons in particular on implant alignment. For this purpose, the use of robot-assisted total knee arthroplasty has become increasingly common in the last 10 years.

**Methods:**

A total of 46 patients (66 knees) who were operated for knee osteoarthritis with Robotic Surgical Assistant (*ROSA, Zimmer-Biomet, Warshaw, Indiana, USA*) between 2021 and 2023 were included in the study. Preoperative planning and intraoperative incision time, total surgical time, range of motion and follow-up time recorded. Oxford knee scores and knee society scores (KSS) of the patients were compared before and after surgery. At the last follow-up Forgotten Joint Score and the sagittal and coronal plane alignments were evaluated.

**Results:**

Preoperative mean Oxford score of the right knee of the patients was 18.5 ± 3.2, post-surgery mean Oxford score progressed to 43.5 ± 2.2. While the preoperative left knee Oxford score of the patients was 16.9 ± 2.3, the mean left knee Oxford score improved to 43.4 ± 2.2 postoperatively. The mean KSS score of the patients’ right knee preoperatively was 49.7 ± 3.5, and progressed to 89.2 ± 4.7 postoperatively. While the preoperative mean left knee KSS score of the patients was 46.5 ± 4.3, the mean KSS score improved to 89.8 ± 3.2 postoperatively. The mean Forgotten Joint Score of the left knee at the last follow-up of the patients was 77.4 ± 3.8, while the mean Forgotten Joint Score of the right knee was 75.4 ± 5.9.

**Conclusion:**

The results of ROSA-supported knee arthroplasty found to be functionally successful.

## 1. Introduction

Total knee arthroplasty is a basic surgical procedure performed in patients who cannot be treated non-surgically in the end-stage knee osteoarthritis.^[1,2]^ Despite the development of surgical techniques in total knee arthroplasty, as well as the advancement of technology, pain complaints continue in 20% of patients. It has been emphasized in the literature that this may be related to ligament imbalance or implant positioning.^[3]^ In total knee arthroplasty, mechanical alignment has been applied in the last 30 years and the hip-knee-ankle (HKA) angle has been adopted as a neutral position. It is thought that the placement of the implants perpendicular to the mechanical axis as neutral alignment and inconsistency with the lower extremity alignment of the patient before the surgery affect the survival of the implants and the results.^[4–7]^ For this purpose, the idea of kinematic alignment gained value, and the patient-specific use of the implant alignment in the sagittal and coronal planes would affect the results.

Kinematic alignment, which was popularized by Howell et al in 2006, has been applied to the present day.^[8]^ The authors who accept this view advocate the idea that optimal connective and soft tissue balance is achieved by placing the implants in accordance with the patient anatomy.^[9,10]^ However, this alignment is known to cause stress on the tibiofemoral and patellofemoral joints.^[11]^

With the development of robotic surgeries in other branches of medicine, it has started to take place in arthroplasty surgeries since 1990. Following the technological developments, integration of computers into robotic systems has enabled these instruments to support surgery. These robotic devices are widely used in orthopedic surgery today as autonomous and semi-autonomous systems. In the surgical application performed with these robotic systems, the positioning of the implants in the sagittal and coronal planes and the ligament balance are optimally provided, and it is argued that the functional results are better.^[12]^

In this era of surgical innovation, the Robot-Assisted Knee Arthroplasty System, known as ROSA, has emerged as a prominent player in the quest for enhanced surgical outcomes. ROSA (*Zimmer-Biomet, Warshaw, Indiana, USA*) is a semi-autonomous robotic system that was first approved by the FDA in 2019 and is still used today. This system enables the surgeon to place the components in the optimum position to adjust the alignment of the components in the sagittal and coronal planes by making minimal bone cuts with loading the preoperative length graphs into the system or with the trackers placed intraoperatively.^[13]^ In this study, we aimed to present the clinical results and implant alignments of ROSA-supported total knee arthroplasty performed in our clinic since 2021.

## 2. Materials and methods

Approval of the local ethics committee was obtained before data collection (Liv Private Hospital 2023/011). A total of 65 patients who underwent ROSA robot-assisted knee arthroplasty for end-stage knee osteoarthritis between 2021 and 2023 were included in the study retrospectively. Patient data were accessed from the hospital automation system. Patients who did not come for their last control and whose data could not be reached were excluded from the study. The remaining 46 patients and total 66 knees were included in the study. Demographic data of the patients such as age, gender, operated side, surgical planning time, total surgery time, total intraoperative time and follow-up were recorded. Simultaneously, preoperative Oxford knee score, Knee Society Score (KSS), HKA, varus - valgus and flexion angles of the femoral component, varus - valgus and slope angles of the tibial component, and knee range of motion data were recorded. Component sagittal and coronal alignments, operation time, and intraoperative joint range of motion were recorded from the ROSA computer system on a patient basis. The duration of the operation was determined as the time from the beginning use of the robot system to the placement of the original components with cement and evaluation from the ROSA system. Surgery, planning and intraoperative time were evaluated for both knee separately in patients who underwent bilateral knee arthroplasty. The patients were called for their final controls and postoperative Oxford knee score, KSS score, and Forgotten Joint Score were recorded.

### 2.1. Operative details

**Figure 1.**
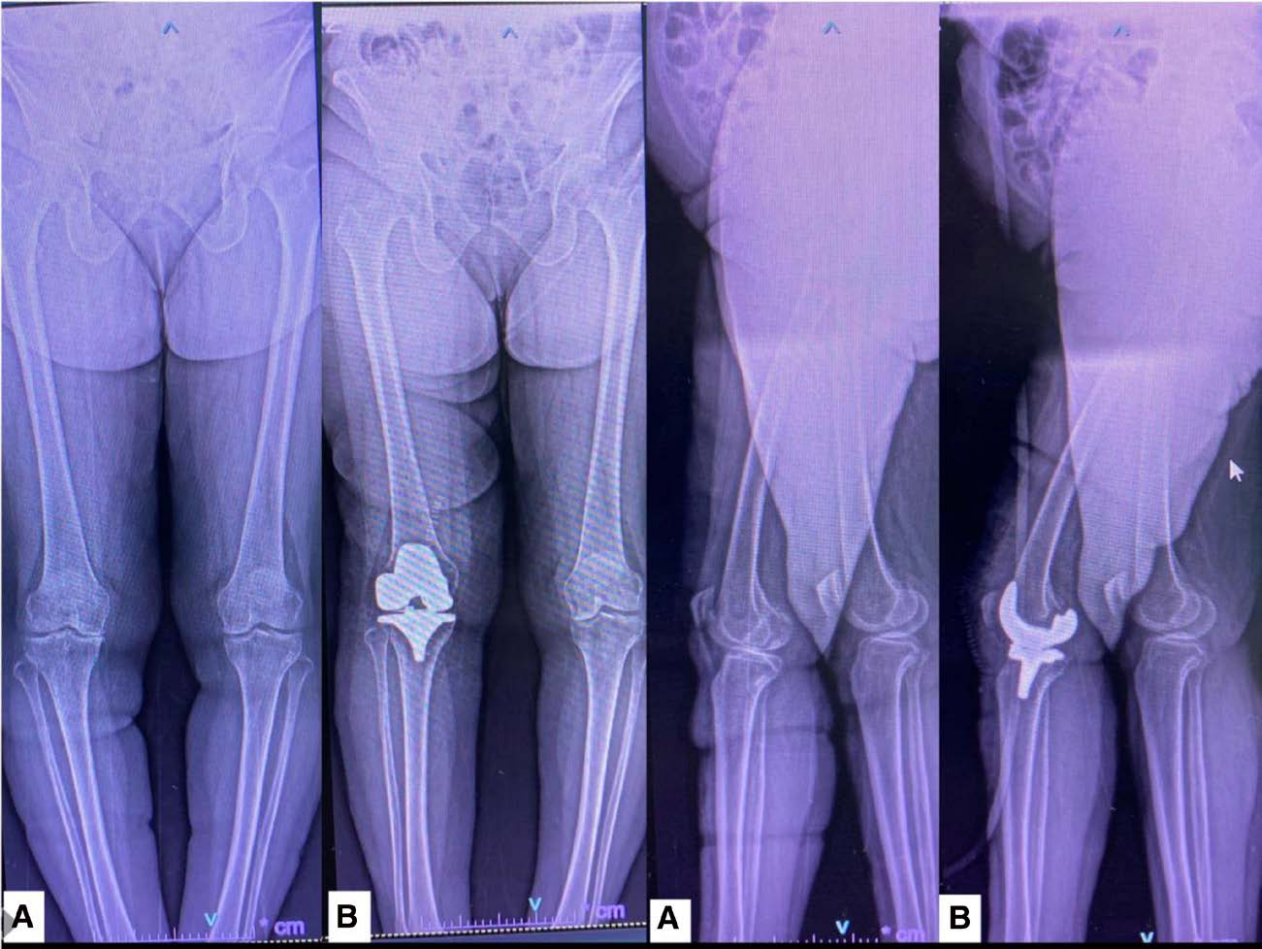
(A) Preoperative AP view X-ray of the patient. (B) Post-operative AP view X-ray of the patient. (c) Preoperative Lateral view of the patient. (d) Post-operative Lateral view of the patient.

All patients were operated by surgeons trained in ROSA robot-assisted knee arthroplasty. The same surgical procedure was performed in all patients. After the tourniquet was applied to all patients and sterile conditions were provided, 2 3.2 mm pins were placed proximally into the femoral shaft and 2 3.2 mm pins were placed approximately 5 cm distal to the tibial tuberosity via a midline stab incision. Femoral and tibial trackers were placed on these pins and after the median parapatellar arthrotomy was performed, a cemented Vanguard (*Zimmer-Biomet, Warshaw, Indiana, USA*) implant was applied within the principles of kinematic alignment.^[14]^ The operation was performed as described by Klein et al for ROSA robot-assisted knee arthroplasty. No patellar component was placed in the patients. All patients were mobilized with the aid of a walker on the first day. Low molecular weight heparin was used for 1 month in all patients. In the postoperative period, Continuous Passive Motion was used for 10 days in all patients (Fig. 1).

### 2.2. Statistical analysis

Statistical analysis was performed using the Statistical Package for the Social Sciences (SPSS) 22 package program. Categorical data were expressed as mean and standard deviation. Comparison of categorical data was evaluated with paired sample t test. The results were evaluated at the 95% confidence interval, and the significance was evaluated at the *P* < .05 level.

## 3. Results

Of the 46 patients included in the study, 7 were male and 39 were female. The mean age of the patients was 57.8 ± 4.1. Right knee arthroplasty was performed in fourteen patients, left knee arthroplasty in twelve patients, and bilateral knee arthroplasty in twenty patients. The mean follow-up period was 17.1 ± 5.1 months (Table 1).

**Table 1:** Demographic data of the patients.

		Age	Side		
		Mean Standard Deviation	Right	Left	Bilateral
Gender	Male	55.2 ± 7.3	3	4	0
	Female	58.3 ± 7	11	8	20
	Total	57.8 ± 7.1	14	12	20

The mean extension angle of the patients who underwent right knee arthroplasty was 2.9 ± 1.9 degrees, while the flexion angle was 122.8 ± 14.6 degrees. The mean extension angle of the patients who underwent left knee arthroplasty was 3.6 ± 2.4 degrees, while the flexion angle was 125.2 ± 12.3 degrees. When the preoperative Oxford knee score and postoperative knee scores of the patients included in the study were compared, there was a statistically significant difference (*P* = .00). When the patients’ preoperative KSS score and postoperative KSS score were compared, there was a statistically significant difference (*P* = .00). The mean Forgotten Joint Score of the left knee at the last follow-up of the patients was 77.4 ± 3.8, while the mean Forgotten Joint Score of the right knee was 75.4 ± 5.9 (Table 2).

**Table 2:** Sagittal and coronal alignment of the components.

		PFKKA (varus)	PFKSA (flexion)	PTKKA (varus)	PTKSA (posterior slope)	Pre-op Right HKA	Post-op Right HKA	Pre-op Left HKA	Post-op Left HKA
		Mean Standard Deviation							
SİDE	Right	1.4 ± 0.8	3 ± 0.6	1.3 ± 0.7	4 ± 1.8				
	Left	1.4 ± 0.9	2.9 ± 0.9	1.3 ± 0.9	3.6 ± 0.9	10 ± 3.8	2.5 ± 1.1	10.1 ± 3.7	2.7 ± 0.9

HKA *=* Hip and Knee Angle, PFKKA *=* planned femoral component coronal alignment, PFKSA *=* planned femoral component sagittal alignment, PTKKA *=* planned tibial component coronal alignment, PTKSA *=* planned tibial component sagittal alignment

**Figure 2.**
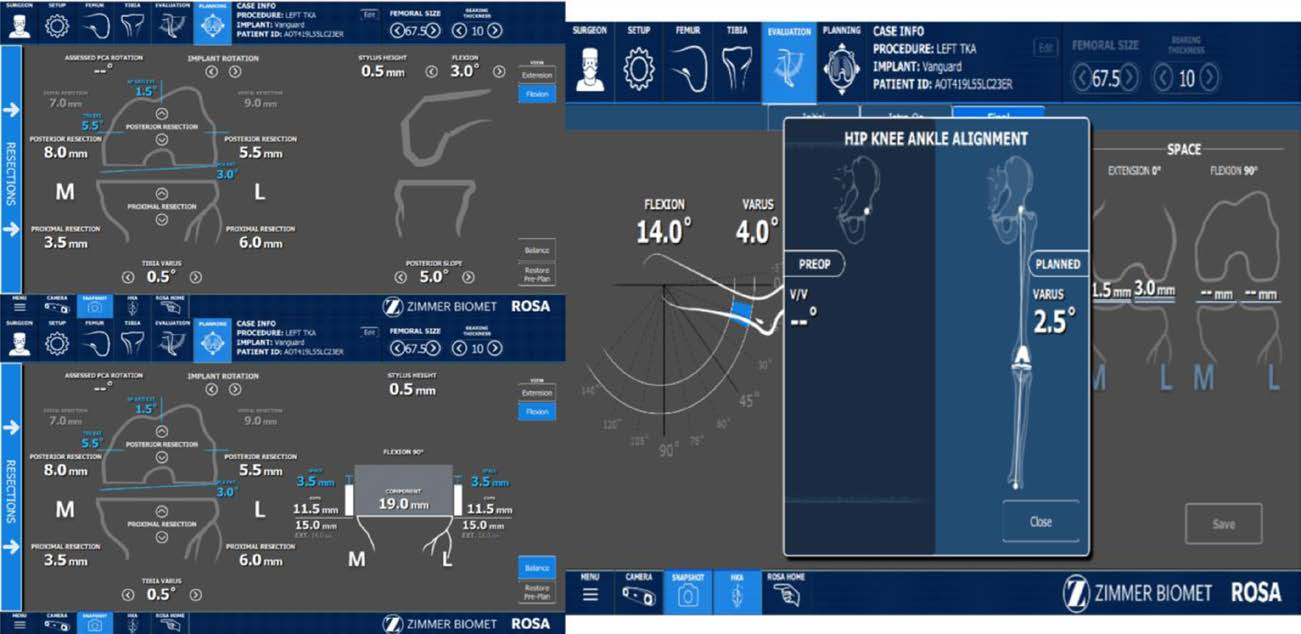
Intraoperative planning with ROSA of the patient. ROSA = Robot-assisted Knee Arthroplasty System.

The mean angle of the femoral component of the right knee was 1.4 ± 0.8 degrees in the coronal plane, and the mean angle in the sagittal plane was 3 ± 0.6 degrees of flexion. While the mean angle of the tibial component of the right knee was 1.3 ± 0.7 varus in the coronal plane, the mean slope angle was 4 ± 1.8 degrees in the sagittal plane. The mean angle of the femoral component of the left knee was 1.4 ± 0.9 degrees in the coronal plane, and the mean angle in the sagittal plane was 2.9 ± 0.9 degrees of flexion. While the mean angle of the tibial component of the left knee was 1.3 ± 0.9 varus in the coronal plane, the mean slope angle was 3.6 ± 0.9 degrees in the sagittal plane (Fig. 2).

The mean HKA angle of the patients who underwent right knee arthroplasty was 10 ± 3.8 degrees preoperatively, and 2.5 ± 1.1 degrees postoperatively, while the mean HKA angle of the patients who underwent left knee arthroplasty was 10.1 ± 3.7 degrees preoperatively and 2.7 ± 0.9 degrees postoperatively. It was determined that the HKA angle was placed in the varus in all patients (Table 3).

**Table 3:** Patients’ preoperative and postoperative functional scores.

		Preoperative KSS(α)	Postoperative KSS(β)	Preoperative oxford score(γ)	Postoperative oxford score(δ)	Forgetten joint score
		Mean standard deviation	Mean standard deviation	Mean standard deviation	Mean standard deviation	Mean standard deviation
Side	Right	49.7 ± 3.5	89.2 ± 4.7	18.5 ± 3.2	43.5 ± 2.2	75.4 ± 5.9
	Left	46.5 ± 4.3	89.8 ± 3.2	16.9 ± 2.3	43.4 ± 2.2	77.4 ± 3.8

While the mean total surgical time of the patients who underwent right knee arthroplasty was 58.5 ± 18.6 minutes, the surgical planning time was 18.2 ± 8.9 minutes and the bone cut time was 13.4 ± 2.8 minutes. While the mean total surgical time of the patients who underwent left knee arthroplasty was 63 ± 17.8 minutes, the surgical planning time was 20.5 ± 10.5 minutes and the bone cut time was 13.7 ± 5.7 minutes (Table 4).

**Table 4:** Operative time (min).

	Total surgical time	Planning	Bone cutting time
	Mean standard deviation		
Right	58.5 ± 18.6	18.2 ± 8.9	13.4 ± 2.8
Left	63 ± 17.8	20.5 ± 10.5	13.7 ± 5.7

One patient included in the study developed an infection that did not respond to antibiotic treatment in the postoperative period. The patient was re-operated in the acute period, and debridement with implant retention was performed. After the patient infection continued after Debridement, antibiotics and implant retention procedure, a 1-stage revision knee arthroplasty was performed. Deep vein thrombosis was detected in another patient despite using thromboprophylaxis in the postoperative period. This patient was treated by anti-thrombolytic treatment. No complications were detected in other patients.

## 4. Discussion

ROSA robot-assisted knee arthroplasty was first used in Australia in 2018, and was approved by the FDA (US Federal Food and Drug administration) in 2019 and started to be used all over the world. One of the most important features that distinguishes it from other robotic systems is that computerized tomography (CT) imaging is not required in the preoperative period. ROSA can be used in 2 ways. First, X-rays are taken with a special x-ray marker in the preoperative period and then intraoperative landmarks are marked, and secondly, intraoperative landmarks are marked after placing trackers on the femur and tibia during the operation. Thus, it prevents patients from receiving unnecessarily high amounts of radiation.^[14]^ It has been stated in the literature that, the use of robotic systems in total knee arthroplasty improve implant positioning, less soft tissue dissection is required, minimal bone incisions provide optimal ligament imbalance, and the patient rehabilitation is becoming easier and faster.^[15,16]^ In addition, less bleeding due to minimal soft tissue manipulations and shorter hospital stay and greater range of motion in the early period are other advantages of robotic knee arthroplasty.^[17]^ It was emphasized in the literature that patients who underwent ROSA-supported knee arthroplasty had similar results.^[18]^ ROSA, which allows the positioning of the femoral and tibial components in the sagittal and coronal planes, provides the optimal ligament balance and allows the placement of the components with 99% accuracy.^[14]^

Although there are many studies in the literature on knee functional scores in robotic assisted non-ROSA knee arthroplasty, there are limited studies with ROSA. In a multicentric study of 1016 patients, Khan et al compared the functional results of ROSA and total knee arthroplasty performed with conventional method, and they found that patients who were operated on with ROSA at the end of the first 6 months and 1 year were able to mobilize faster, and their KOOS-JR scores were higher.^[19]^ In another study conducted by Mancino et al, with 173 patients, the patients who had undergone total knee arthroplasty in 2 different ways as ROSA and imageless navigated procedure (NTKA, iAssist Knee, Zimmer, Warsaw, IN) were divided into 2 groups and their functional scores at the end of 1 year were compared. At the end of the study, they found that patients who were operated with ROSA had higher KSS score and range of motion. The same study found the Forgotten Joint Score to be 72.6 ± 22.3 at the end of 1 year.^[20]^

One of the important problems in robotic surgeries is the concern about the prolongation of the surgical time. However, in the study conducted by Kayani et al comparing the total surgical time of conventional knee arthroplasty and robot-assisted knee arthroplasty, although it was longer than the conventional method in the first 10 cases, they did not find a statistically significant difference when they compared the average total time.^[21]^ In another study by Vanlommel et al in which they compared the results of ROSA knee arthroplasty with the conventional method, they found the mean total surgical time of knee arthroplasty performed with ROSA to be 91.3 ± 11.9 minutes, and 73.3 ± 11.3 with the conventional method.^[22]^ In our study, we found the mean total surgical time to be 58.5 ± 18.6 minutes for the right knee and 63 ± 17.8 for the left knee. This period was shorter than in the literature.

In the study conducted by Rossi et al with ROSA-supported knee arthroplasty, they evaluated 75 patients and found the mean femoral component flexion angle as 2.7 ± 1, varus angle as 1.3 ± 1, tibia varus angle as 0.5 ± 0.7, and tibial slope angle as 3 ± 0.3. They calculated the planned mean HKA angle as 178.2 ± 1.2 in all patients.^[23]^ In our study, we found the angles to be compatible with the literature.

There were some limitations in our study. First of all, since the post-operative length radiographs were not available in all patients, the angles of the planned incision and the components placed on the radiographs could not be compared. Secondly, the evaluation of comparative results with the conventional method will be more accurate in terms of evaluating the results of robotic surgery. However, since the use of ROSA robotic knee arthroplasty was started in 2019, the limited number of studies in the literature makes our study valuable.

In conclusion, ROSA-supported knee arthroplasty is a very successful surgical method in terms of functional results when the first-year results are evaluated. This system can place components with 99% accuracy in the sagittal and coronal planes, as stated in the literature and similar to our evaluation. The publication of long-term results in the future will determine the future use of this robotic system.

## Acknowledgments

The authors thank to all hospital workers for their high efforts.

## Author contributions

**Conceptualization:** Yunus Demirtas, Abdulsamet Emet, Gokhan Ayik.

**Data curation:** Yunus Demirtas, Abdulsamet Emet.

**Formal analysis:** Abdulsamet Emet.

**Methodology:** Abdulsamet Emet, Gokhan Ayik, Gokhan Cakmak, Ertugrul Aksahin, Levent Celebi.

**Supervision:** Abdulsamet Emet, Gokhan Cakmak, Ertugrul Aksahin, Levent Celebi.

**Writing – original draft:** Yunus Demirtas, Abdulsamet Emet, Ertugrul Aksahin, Levent Celebi.

**Writing – review & editing:** Yunus Demirtas, Abdulsamet Emet, Gokhan Ayik, Gokhan Cakmak, Ertugrul Aksahin, Levent Celebi.
